# A gene-based high-resolution comparative radiation hybrid map as a framework for genome sequence assembly of a bovine chromosome 6 region associated with QTL for growth, body composition, and milk performance traits

**DOI:** 10.1186/1471-2164-7-53

**Published:** 2006-03-16

**Authors:** Rosemarie Weikard, Tom Goldammer, Pascal Laurent, James E Womack, Christa Kuehn

**Affiliations:** 1 Forschungsinstitut für die Biologie landwirtschaftlicher Nutztiere (FBN), 19196 Dummerstorf; Germany; 2 Laboratoire de Génétique et de Cytogénétique, INRA, Jouy-en-Josas, 78350, France; 3 Texas A&M University, College Station, 77843-4467, USA

## Abstract

**Background:**

A number of different quantitative trait loci (QTL) for various phenotypic traits, including milk production, functional, and conformation traits in dairy cattle as well as growth and body composition traits in meat cattle, have been mapped consistently in the middle region of bovine chromosome 6 (BTA6). Dense genetic and physical maps and, ultimately, a fully annotated genome sequence as well as their mutual connections are required to efficiently identify genes and gene variants responsible for genetic variation of phenotypic traits. A comprehensive high-resolution gene-rich map linking densely spaced bovine markers and genes to the annotated human genome sequence is required as a framework to facilitate this approach for the region on BTA6 carrying the QTL.

**Results:**

Therefore, we constructed a high-resolution radiation hybrid (RH) map for the QTL containing chromosomal region of BTA6. This new RH map with a total of 234 loci including 115 genes and ESTs displays a substantial increase in loci density compared to existing physical BTA6 maps. Screening the available bovine genome sequence resources, a total of 73 loci could be assigned to sequence contigs, which were already identified as specific for BTA6. For 43 loci, corresponding sequence contigs, which were not yet placed on the bovine genome assembly, were identified. In addition, the improved potential of this high-resolution RH map for BTA6 with respect to comparative mapping was demonstrated. Mapping a large number of genes on BTA6 and cross-referencing them with map locations in corresponding syntenic multi-species chromosome segments (human, mouse, rat, dog, chicken) achieved a refined accurate alignment of conserved segments and evolutionary breakpoints across the species included.

**Conclusion:**

The gene-anchored high-resolution RH map (1 locus/300 kb) for the targeted region of BTA6 presented here will provide a valuable platform to guide high-quality assembling and annotation of the currently existing bovine genome sequence draft to establish the final architecture of BTA6. Hence, a sequence-based map will provide a key resource to facilitate prospective continued efforts for the selection and validation of relevant positional and functional candidates underlying QTL for milk production and growth-related traits mapped on BTA6 and on similar chromosomal regions from evolutionary closely related species like sheep and goat. Furthermore, the high-resolution sequence-referenced BTA6 map will enable precise identification of multi-species conserved chromosome segments and evolutionary breakpoints in mammalian phylogenetic studies.

## Background

In October 2004, the initial draft of the bovine genome sequence using a whole genome shotgun strategy has been released [1,2]. Preliminary assemblies of the current bovine genome sequence update representing a 6× coverage have been established using whole genome shotgun sequences (WGS) and BAC end sequences (BES) on bovine chromosomes and were announced in October 2005 (Pre! Ensembl (Btau 2.0) and NCBI Bos taurus genome mapview (build 2.1) [3,4]).

A high-resolution physical map will provide a basic framework to assist the final high-quality sequence assembly and annotation process of the bovine genome for anchoring genes, anonymous loci, WGS, BES, and preliminary contigs along bovine chromosomes. Subsequently, the availability of the complete architecture of bovine chromosomes will facilitate positional cloning efforts focusing on targeted identification of the causative sequence variation underlying quantitative trait loci (QTL) [5].

In order to construct a high-resolution map for a specific chromosome or targeted chromosomal region, comparative mapping information from maps of the annotated human and mouse genomes can be utilized efficiently. The location of bovine loci that are homologues of human genes may be predicted from the current knowledge about the conservation of synteny between genomes, but has to be actually proven by direct mapping on the bovine genome. However, the successful use of genome information from sequence-ready maps relies upon the precise identification and characterization of segments of conserved synteny, gene order, and evolutionary breakpoints in the bovine genome. Radiation hybrid (RH) mapping has been shown to be a powerful tool to integrate comparative genome data with information from existing bovine genetic and physical maps to generate high-resolution maps (e.g., [6]). Considering all RH panels available for the bovine genome, the 12,000 rad whole genome RH panel (12 k WG-RH, [7]) has been shown to have the high mapping power required to create accurate maps of a highly elevated resolution in defined small chromosomal regions [8-11].

In our study, bovine chromosome 6 (BTA6) was chosen as a candidate chromosome, because a number of different QTL for various phenotypic traits, including milk production, functional, and conformation traits in dairy cattle as well as growth and body composition traits in meat cattle, have been mapped consistently in the middle region of this chromosome (e.g., summarized at [12,13]). Although, initial results focusing on the candidate genes underlying the QTL for milk production traits at the molecular level have been reported recently [14-18], further structural and functional research is required to dissect the different QTL at the gene-based level.

Previous comparative studies based on fluorescence *in situ *hybridization (FISH; e.g., [19]) and RH mapping (e.g., [6,20,21]) revealed that the region associated to the indicated QTL on BTA6 corresponds to two segments of conserved synteny on human chromosome 4 (HSA4). However, multiple inter- and intrachromosomal micro-rearrangements with respect to orthologous human chromosomes as reported for several bovine chromosomes (e.g., BTA13: [8], BTA7: [22], BTA27: [23]) could not be excluded given the data currently available for BTA6. Consequently, to enable fine mapping of QTL and final identification of causal genes underlying the QTL within small chromosome segments of BTA6, the content and order of genes within the syntenic regions need to be defined and characterized precisely.

Hence, the objective of this study was to construct a high-resolution "gene-rich" RH map for the targeted chromosomal region of BTA6. Based on our first BTA6 RH map [9] and by exploiting the available human and bovine genome sequence information, RH mapping was utilized to link both microsatellite markers and genes into an improved comprehensive, high-resolution map. In order to increase the number of comparative anchor loci on the physical map of BTA6 and to refine boundaries for blocks of conserved synteny, a large number of new genes, which have been derived from the orthologous region of the human genome was mapped onto BTA6. In addition, novel targeted isolated anonymous markers and BES were integrated into the RH map of the BTA6 region.

## Results

### Framework and comprehensive RH map construction

A total number of 237 loci (See Additional file 1) including 115 genes and expressed sequence tags (ESTs) were typed on the cattle-hamster 12 k WG-RH panel [7]. Hundred eleven genes and ESTs have human orthologous loci in the syntenic chromosomal region on HSA4. No significant sequence similarity to human genes and ESTs was found for 4 bovine ESTs (*FBNE2, FBNE3, FBNL089, FBNI011*). For the 237 loci tested, 211 different retention patterns were observed. A total of 191 loci displayed unique retention patterns, whereas 46 shared identical retention patterns with at least one other locus indicating no obligate chromosome breaks between them (see Additional file 2).

**Figure 1:**
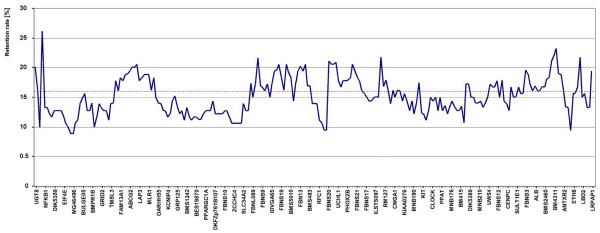
**Histogram of retention frequencies of 237 loci on the defined region of BTA6**. Every fourth locus is given by name on the horizontal axis. The order of loci corresponds to the order in the comprehensive map. The left end is directed to the centromere and the right end to telomere of BTA6. The red horizontal line indicates the mean retention frequency.

The retention frequencies of loci varied from 8.9 % for *CSSM59 *and *PTPN13 *to 26.1 % for *MANBA*, and averaged 15.2 % for all loci. The distribution of the retention frequencies across the chromosomal region covered is shown in Figure 1. Retention pattern did not show a consistent bias along the chromosomal region.

**Figure 2:**
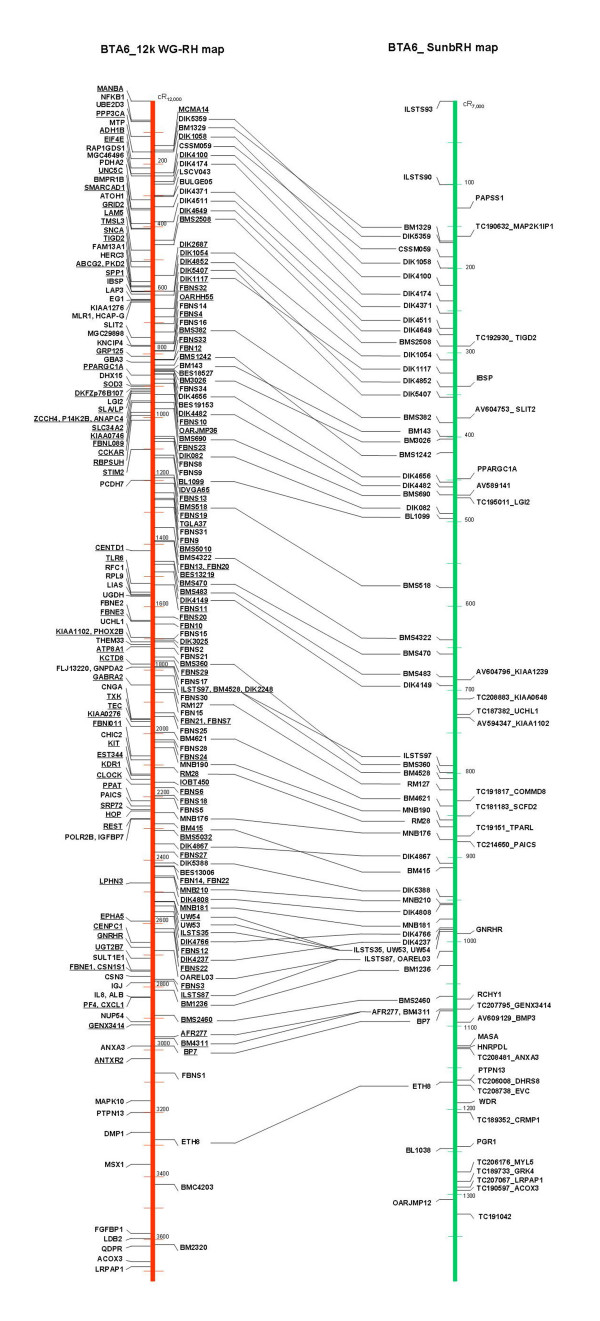
**Alignment of BTA6 radiation hybrid maps**. Left: BTA6 12 k WG-RH map generated in this study. The loci presented on the right side of the map are anonymous markers; the loci displayed on the left side of the map represent genes and ESTs. Underlined loci indicate framework loci. Right: BTA6 SunbRH map [6]. Genes and ESTs are sorted on the right side of the map, whereas on the left side of the map those anonymous markers, which were also typed on the 12 k WG-RH panel, are shown.

The chromosomal region analyzed is represented by one contiguous RH linkage group at twopoint lodscore (LOD) criterion 4 comprising a total of 234 loci and by 3 singletons. The positions of the singletons (*UGT8, FLJ20032 *and *CENPE) *on BTA6 were retrieved from published bovine maps [20,21,24]. At twopoint LOD threshold 6, the large twopoint LOD 4-linkage group is fragmented into one major group containing 228 loci, and one further linkage group consisting of 6 loci. Maximum likelihood multipoint linkage analysis integrated 139 loci (see Additional file 2, highlighted as light green cells) with unique retention pattern into a framework map at multipoint LOD 3 as described in Materials and Methods. On the basis of this framework map encompassing the region between *MANBA *and *ANTRX2*, we sequentially integrated the remaining loci to generate a comprehensive map with the most likely order (Figure 2). The resulting marker order of the comprehensive map spanning 3,685 cR_12,000 _as determined by the maximum likelihood program MAXLIK is in agreement with the one determined by calculating the order with the minimal number of obligate breaks option (MINBRK) and by subjecting the proposed order to a ripple procedure (analogous to the "ripple" function of the RHMAPPER program) along the chromosomal region.

The respective chromosomal region of BTA6 we focused in this study includes the segment between *BM1329 *as the most proximal marker and *BM2320 *as the anchor marker in the distal part. According to the comprehensive genetic map [25], this segment comprises a distance of about 91.87 cM. In comparison to our RH map, a genome ratio of 37.65 cR_12,000_/cM was calculated, which corresponded to the value obtained in our initial study (42.8 cR_12,000_/cM, [8]). Considering the preliminary bovine sequence assemblies of BTA6 provided at the Ensembl and NCBI databases (Btau 2.0 and build 2.1, [26,27]), a ratio of 58.5 cR_12,000_/Mb was inferred for this chromosomal region. The average inter-loci interval on the targeted BTA6 region covered on our RH map is 17.8 cR_12,000 _corresponding to approximately 300 kb.

### Identification of whole genome contig sequences specific to BTA6

The comprehensive comparison of our high-resolution RH map with the genome sequence map of BTA6 required the identification of additional sequence contig scaffolds of the existing 6× coverage draft of the bovine genome sequence specific to our BTA6 region. Therefore, all sequences (136 loci) integrated in our high-resolution BTA6 RH map but not placed on the current NCBI sequence assembly map of BTA6 (build 2.1, [27]) were used to screen the bovine genome sequence resources [28]. A total of 73 loci could be assigned to sequence contigs, which were already assigned to BTA6 (see Additional file 2, labeled in bold red). For 43 loci, we detected corresponding sequence contigs, which were not yet placed on the bovine genome (see Additional file 2, labeled in blue). Furthermore, 7 loci (*PPP3CA, ADH1B, PDHA2, OARHH55, ALB, EG1, FGFBP1*) were found on sequence contigs, which had been assigned to other chromosomes than BTA6 (see Additional file 2, highlighted as yellow cells). For a total of 13 loci, no sequence contigs were found within the available bovine sequence resources.

### Generation of the comparative map

With the aim to construct a high-resolution multi-species comparative RH map, we started with 52 genes and ESTs previously assigned to BTA6 [6,9,21,29]. Furthermore, we added bovine genes inferred from their human orthologs localized in the syntenic chromosome segment of HSA4p16.3-HSA4q26, for which corresponding bovine mRNA sequences were known [30]. To further increase the number of mapped coding sequences on BTA6 as comparative anchor loci, we started again from the syntenic human chromosomal region of HSA4 by screening sequences of known and predicted human genes from this region using the BLASTN algorithm against the cattle EST database [28]. This comparative *in silico *mapping approach is the targeted version of the COMPASS mapping procedure (e.g., [31]), which utilizes the human-cattle comparative mapping knowledge for predictive positioning of unknown bovine ESTs on the bovine genome. Following this targeted mapping approach, we identified a total of 60 new bovine gene loci (see Additional file 1, highlighted in red) derived from human gene sequences with known positions on HSA4, and verified and localized them on the comprehensive RH map of BTA6. In addition, 3 bovine chromosome region-specific ESTs (unpublished results) were integrated on our high-resolution RH map of BTA6.

A further approach focused on linking 126 anonymous loci mapped on our RH map of BTA6 (microsatellite markers, novel targeted isolated markers as sequence-tagged sites (STS), BES, and ESTs) with the syntenic HSA4 region by merging sequence and mapping information. Therefore, these sequences were applied for direct similarity search with the human genome sequence using the BLASTN program [32] to find positions of orthology on the syntenic chromosome HSA4. For 44 out of these anonymous markers, orthologous sequences localized on HSA4 were identified (35 %). Placement of these 44 anonymous sequences on HSA4 was derived from the positions of human BAC contigs harboring sequence motifs homologous to the targeted bovine sequences (see Additional file 2, highlighted in bold blue). Homology search of all 126 anonymous sequences against the whole human genome did not reveal any unique and significant homologous region on other chromosomes than HSA4.

To better define the BTA6-HSA4 comparative map, the bovine genomic sequences (WGS contigs) containing the anonymous marker sequences were used as intermediary sequences between bovine anonymous loci and the human genomic sequence as described in Methods. Thus, comparative positions of anonymous markers without direct human alignments were established by performing sequence similarity search on the human genome sequence with bovine WGS contig sequences containing the targeted marker sequences. Including the information from the intermediary alignment procedure, 72 additional anchor points could be localized on HSA4 (see Additional file 2, identified HSA4 BACs are indicated as underlined blue). Ten marker sequences could not be anchored on the human genome sequence by this *in silico *mapping procedure.

**Figure 3:**
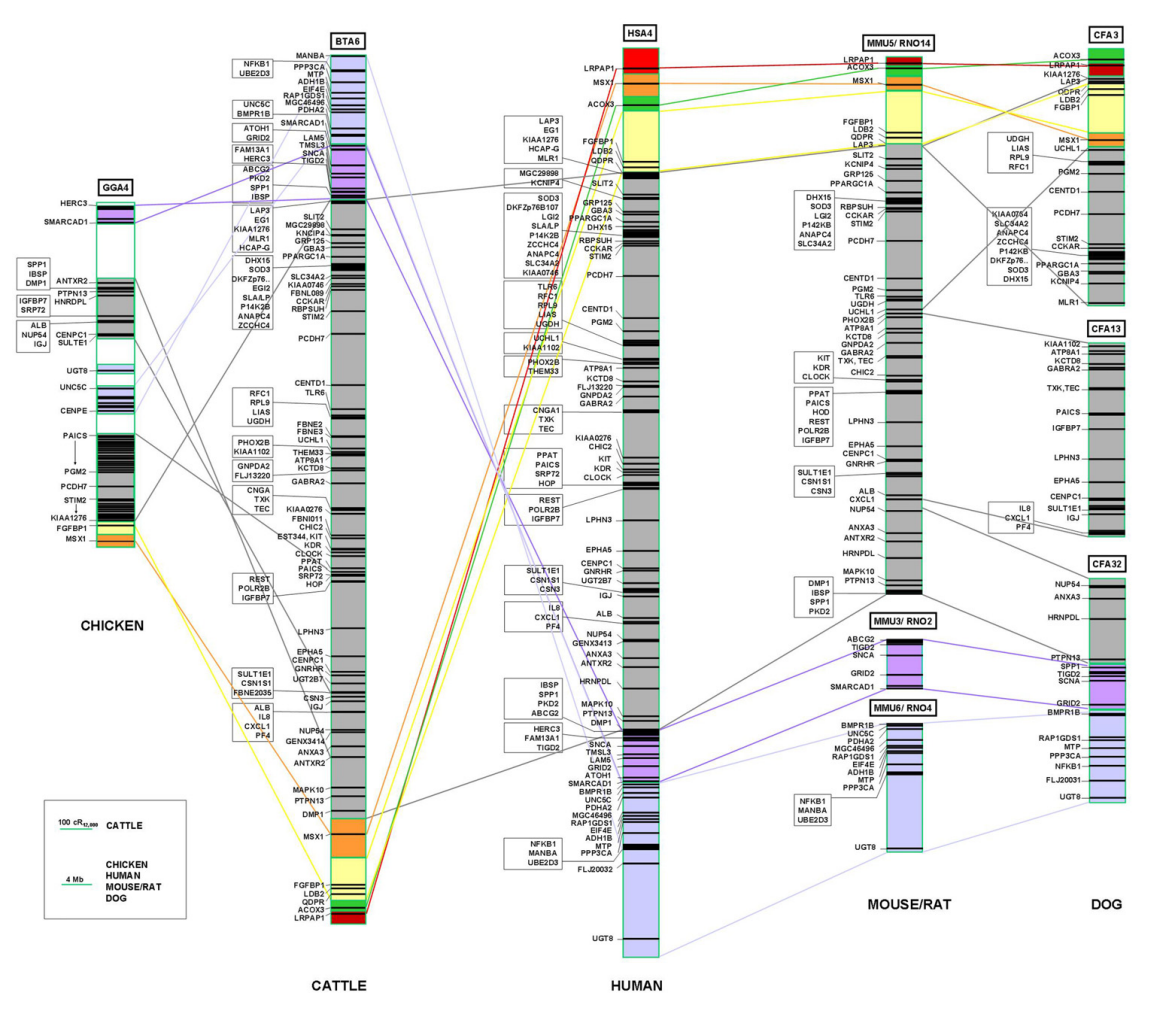
**Comparison of gene order on the 12 k WG-RH map of BTA6 with the gene architecture on syntenic chromosomal regions of human, mouse, rat, chicken, and dog**. For comparison of gene positions mapped on the BTA6 12 k WG-RH map with corresponding genome map positions on orthologous chromosomal regions of human, mouse, rat, chicken, and dog, gene mapping information was retrieved from the species genome sequence maps available at the NCBI mapviewer database [33]: human chromosome 4 (HSA4, NCBI build 35.1), mouse chromosomes 5, 6, and 3 (MMU5, 6, and 3; NCBI build 34.1), rat chromosomes 14, 2, and 4 (RNO14, 2, and 4; NCBI build 3.1), dog chromosomes 3, 13, and 32 (CFA3,13 and 32, NCBI build1.1), and chicken chromosome 4 (GGA4, NCBI build 1.1). Horizontal black lines on species sequence maps indicate positions of loci on the chromosomes, black blocks on the maps represent high gene density gene-covered intervals. Loci with close mapping positions are depicted in boxes. Distances of loci on sequence maps of human, mouse, rat, dog, and chicken are based on identical Mb scale. Distances on the BTA6 12 k WG-RH map developed in this report are given in cR. Different colored blocks and lines indicate regions of interchromosomal breaks observed between HSA4 and syntenic multi-species chromosomes. White blocks on GGA4 indicate chromosomal regions syntenic to other human chromosomes.

Finally, the cattle-human comparative map (Figure 3) of the defined chromosomal region on BTA6 was generated by aligning the RH map established in this study with the orthologous region of HSA4 taking the human genome sequence assembly as a reference. Physical map positions of human genes and ESTs for the construction of the comparative chromosomal map based on the coordinates of the human genome sequence (NCBI build 35.1). For comparison of the chromosomal organization of BTA6 to equivalent chromosomes from other species, gene mapping information of genes on orthologous chromosomal regions [mouse chromosomes 5, 6 and 3 (MMU5, 6, and 3; NCBI build 34.1), rat chromosomes 14, 2, and 4 (RNO14, 2, and 4; NCBI build 3.1), dog chromosomes 3, 13, and 32 (CFA3,13 and 32, NCBI build1.1), and chicken chromosome 4 (GGA4, NCBI build 1.1)] was retrieved from the species genome sequence maps available at the NCBI mapviewer database [33]. The comparative map displaying the gene order in the orthologous chromosomal regions cross-linking our bovine comprehensive 12 k WG-RH map of BTA6 and corresponding chromosomes from multi-species genomes is presented in Figure 3.

## Discussion

### The high-resolution 12 k WG-RH map for BTA6 and comparison with genetic and physical maps

High-resolution comparative mapping in combination with sequencing information can be utilized efficiently not only to create a sequence-ready chromosome map, but also to contribute to the selection of positional and functional candidate genes within chromosomal regions containing genetic variability affecting phenotypes of physiological and economic importance in cattle. For targeted comparative mapping studies in species with lacking or incompletely annotated genomes like the bovine, the availability of the complete architecture of human and mouse genomes provides an excellent resource employing the information from gene-rich maps of the human and mouse genomes in combination with high-resolution synteny RH mapping. This strategy was applied in our study on BTA6 to powerfully accelerate the research on genes associated with QTL for lactation and growth related traits mapped on this chromosome.

The resolution and gene density of the current whole-genome cattle RH maps [6,20,21,34] is often inadequate either to support the assembly of the bovine genome sequence or for the molecular dissection of complex traits in cattle. On BTA6, the proportion of bovine genes that have been mapped is relatively small and unevenly distributed. To overcome these limitations and to construct an improved gene-based map, our aim was to enrich previous marker-dominated maps by integrating available information from anchored genes and by filling in with targeted selected loci to bridge existing gaps. Therefore, comparative alignment of the human sequence of the orthologous chromosome was utilized for *in silico *identification of bovine ESTs specific for the selected chromosome region of BTA6. Furthermore, an increase in density of loci on the BTA6 RH map was achieved by targeted integration of novel markers from a microdissection library [35] and the high-density bovine genetic map [25], particularly in intervals with poor marker coverage.

Out of a total of 237 loci, 234 loci were used to construct an integrated RH map of high-resolution for the targeted chromosomal region of BTA6 using a 12 k WG-RH panel. The average retention frequency of 237 BTA6 loci typed was 15.2 %. This value is in the same magnitude as the frequencies of 16 % found for BTA6 loci in the bovine 5 k WG- RH panel [21] and 15.2 % reported in the 7 k WG-SunbRH panel [6].

Using RH analysis we have included known genes and ESTs from currently available BTA6 maps and added a total of 63 novel genes and ESTs accurately in relation to the position of known loci onto the map. Hence, the density of genes and ESTs integrated on our RH map exceeds the density on available bovine genetic and physical maps of the respective region of BTA6 substantially.

The order of loci determined in our high-resolution, gene-rich map for the targeted BTA6 region is generally consistent with that reported on previous published RH [6,21] and linkage maps [25,36]. Compared to the SunbRH map [6], local inversions of loci pairs were found only for 8 placements within small chromosomal segments (Figure 2). These discrepancies observed between adjacent loci in very close positions could be possibly due to the resolution limit of the RH panels used. Major differences were only detected when comparing the order of loci in our RH map to that in the second BTA6 linkage group of the second generation comprehensive map [21]. In contrast to the latter map, the interval including *SPP1, ABCG2, HERC3*, and *FAM13A1 *is positioned proximal to *IBSP *in a reverse order in our RH map. This placement is in agreement with recently published data [14,16]. Taken together, these two reports and our data provide strong support for the proposed genomic structure of the chromosomal region of BTA6. The genomic structure of this chromosomal region is of special interest, because recently, two different candidate genes for a QTL with effects on milk production traits have been reported there [14,17], which requires further detailed structural and functional analysis of the corresponding chromosomal area.

To connect our high-resolution 12 k WG-RH map for BTA6 with the existing 6× coverage bovine genome sequence assembly, we compared this map with the sequence map available for BTA6 [26] and conducted additional BLAST sequence similarity search with sequences of loci that were not placed there. A total of 116 new placements of loci onto BTA6 were identified providing 73 new assignments onto known BTA6 contigs and 43 placements to sequence contigs from the pool of unassigned bovine sequence contigs in the NCBI archive. In summary, we found 95 % of all sequences mapped on our RH map to be presented in the current 6× coverage draft of the bovine genome sequence exemplarily underlining its high potential as a framework sequence resource to represent the molecular architecture of the bovine chromosomes.

**Figure 4:**
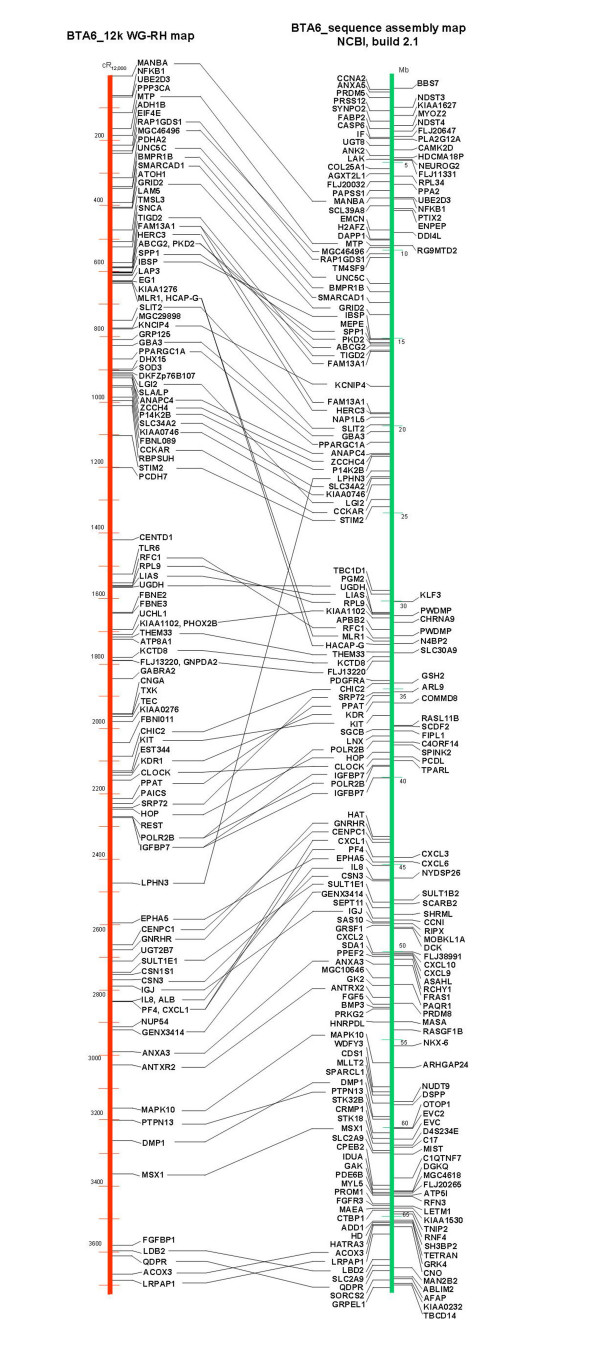
**Comparison of loci order on the 12 k WG-RH map of BTA6 with the preliminary genome sequence assembly of BTA6**. Left: BTA6 12 k WG-RH map developed in this report. Right: Currently available version of the NCBI BTA6 sequence assembly map (build 2.1, [27]). On both maps, only positions of genes are displayed.

Comparing the locus order on our 12 k WG-RH map of BTA6 with the currently available 6× coverage sequence assembly of BTA6 [27] as demonstrated in Figure 4, we found a good overall agreement of both maps, although a number of discordances were observed. These differences in locus order focused as followed indicate regions in the currently available 6× assembly, which have to be considered critically in the course of the curation process to establish the final sequence assembly on BTA6.

Generally, the order of multiple loci on a sequence contig agrees with the order obtained by our RH map (see Additional file 2: e.g., loci on NW_931627.1, NW_931635.1, NW_931636.1, NW_931661.1, NW_931643.1, NW_931673.1, and NW_931689.1). However, orientation of the contigs within the sequence assembly is sometimes opposite to the order of loci in our RH map (NW_931627.1, NW_931635.1, NW_931643.1, NW_931673.1, NW_931701.1, NW_931689.1). Substantial differences in locus order were observed on the current sequence assembly, when the position of sequence contigs is arranged in strong contrast to the placement of the corresponding loci by RH mapping (e.g., for *MLR1 *and *HACP-G *on NW_931678.1; *LPHN3 *on NW_931658.1; *IOBT450 *and *KDR *on NW_931714.1; *PF4 *and *CXL1 *on NW_931715.1). The position of these loci on our RH map is highly supported, because they entered the RH map as framework markers. The arrangement of sequence contigs NW_931635.1, NW_931636.1, and NW_931634.1 observed in the segment between 15 and 20 Mb on the sequence assembly (comprising the loci *SPP1, IBSP, ABCG2, BMS382, HERC3*, and *FAM13A1*) is in contrast not only to our RH map but also to published results based on BAC contigs [14,16] as we discussed above when comparing the existing RH maps. Furthermore, some loci are represented by more than one assignment onto different sequence contigs in the sequence assembly [*FAM13A1 *(15.8 and 19.2 Mb), *POLR2B *(38.1 and 39.3 Mb), *IGFBP7 *(39.4 and 40.1 Mb), *KDR *(35.8 and 44.4 Mb)]. There are 7 loci placed on our RH map (3 of them are framework loci), which are currently assigned to other than BTA6 specific sequence contigs. These positions of loci with questionable placements indicating problem areas in the current assembly have to be refined in further assembly versions.

Screening the resources of bovine genome sequence contigs with sequences mapped on BTA6 but without a placement onto BTA6-specific sequence contigs on the current sequence assembly, we identified 43 new bovine sequence contigs carrying loci, which were mapped on BTA6 (see Additional file 2, highlighted in blue). These new assignments have predominantly filled gaps within subchromosomal regions poorly covered with genes (genome deserts) and could be helpful to guide the validation of the sequence assembly.

Comparing both, our RH map and the sequence assembly map of BTA6 to the loci order on HSA4, the BTA6 RH map shows a lower number of interchromosomal rearrangements with HSA4 than the current sequence contig assembly. As demonstrated in Additional file 2, the order of loci on the RH map is highly consistent within both chromosomal segments syntenic to HSA4: the inversed segment comprising loci from *MANBA *to *IBSP *as well as the segment comprising the loci from *LAP3 *to *DMP1*. There are only two regions in our RH map indicating rearrangements (between *OARHH55 *and *SLIT2 *and within the region comprising loci *DIK082, FBNS8, FBNS9*, and *RBPSUH)*, which have to be reanalyzed in further investigations. In contrast, there appeared a significant difference in the conservation of gene order, when the BTA6 sequence assembly map is compared with HSA4. Considering the high degree of conservation of gene order in syntenic blocks of human and bovine chromosomes as well as in comparison with syntenic blocks in other species (see below in the discussion of comparative maps), the elevated number of intrachromosomal rearrangements suggested by the BTA6 sequence assembly map seems less likely than the gene order determined by our RH map.

However, aligning the positions of loci on our RH map to the preliminary genome sequence assembly of BTA6 revealed an adequate overall agreement in locus order and distances between loci, although a number of inconsistencies have been pointed up because of misassigned contigs and rearrangements. This is supported by a correlation of 0.96 between positions of loci given in cR and Mb (according to Additional file 2).

Based on our 12 k WG-RH map and the genetic map [25], a mean genome ratio of 37.65 cR_12,000_/cM was calculated. The comparison of the resolution of our RH map with the preliminary bovine sequence assembly of BTA6 revealed an estimated ratio of about 58.5 cR_12,000_/Mb. This indicates that one cR on our high-resolution RH map is covered by 17.1 kb on the contig map of BTA6. Aligning the genetic map with the present contig map, a ratio of approximately 643 kb/cM could be deduced, a value deviating from the generally expected ratio 1 Mb/cM.

### The multi-species comparative map

In comparison to previous published bovine-human comparative physical maps of BTA6, we were able to increase the number of anchor points of genes and markers between species. This was the prerequisite to close physical gaps between linkage groups depicted in the comprehensive second generation map [21] as well as the gap between *PPARGC1A *and *IBSP *in the SUNbRH map [6]. Those inter-species breakpoints between human and bovine, which were already reported in our initial map [9], could be refined exactly to be between *LAP3 *and *QDPR *as well as between *IBSP *and *DMP1*. As shown in Figure 3, a highly conserved segment comprising the interval from *LAP3 *to *DMP1 *on HSA4 (17.25 Mb and 88.94 Mb, respectively) is completely maintained on BTA6 without significant intrachromosomal rearrangements. The locus adjacent to *LAP3 *on HSA4, *QDPR *(17.16 Mb on HSA4), which is closely positioned with *LDB2 and FGFBP1 *(16.18 and 15.61 Mb on HSA4), was anchored on the distal part of BTA6 closely to *BM2320 *(Figure 2). The breakpoint on HSA4 and the chromosomal interval, which is attached to QDPR, could be inferred by exploiting the currently available bovine genome sequence assembly. For this purpose, the bovine genomic reference sequence [28] was screened with the sequence of *BM2320*, which detected homology to the bovine BTA6 contig NW_931791. This contig is located adjacent to the contig NW_931792, which is predicted to contain *MAN2B2 (LOC618061)*. *MAN2B2 *is located at 6.7 Mb on HSA4. Thus, considering the comparative *in silico *mapping information, it can be concluded that on BTA6, the chromosomal block containing *MAN2B2 *is attached closely to the breakpoint-containing segment including *QDPR, LDB2*, and *FGFBP1*. Further chromosomal rearrangements compared to HSA4 were observed in the extreme telomeric part of BTA6 around *MSX1*, *ACOX3*, and *LRPAP1 *(4.75, 8.48 and 3.55 Mb on HSA4). In order to resolve this chromosomal region accurately and define the breakpoints, additional loci have to be integrated in the map.

Comparative cross-species analysis of the gene order indicates a highly conserved organization across the human-bovine breakpoints within the interval from *FGBP1-QDPR-LAP3 to DMP1-IBSP-SPP1-PKD2 *on HSA4, MMU5, and RNO14. As outlined above, on BTA6 this interval is shortened to the chromosome segment ranging from *LAP3 *to *DMP1 *due to interchromosomal breaks and rearrangements between *LAP3 *and *QDPR *as well as *DMP1 *and *IBSP*. The general gene order, however, within this ancestral interval *LAP3 to DMP1 *seems to be maintained across species. Comparing the gene positions on the mouse and rat chromosomes syntenic to HSA4, the interval at the breakpoint distal from *PKD2 *is broken again and placed on different chromosomes: the segment from *ABCG2 *to *SMARCAD1 *(likely more exactly *LIM*) on MMU6 and RNO4, respectively, and the segment beginning with *BMPR1B *on MMU3 and RNO2, respectively.

In chicken the whole chromosomal region of the HSA4p16.3-4q26 is maintained on chromosome 4 (GGA4, [37]), although, there is a number of intrachromosomal rearrangements resulting in several individual gene blocks and micro-rearrangements along the chromosome. When compared to HSA4 and BTA6, the syntenic segment on GGA4 revealing the longest stretch of orthologous genes in identical order encompasses the region from *KIAA1276 *to *PAICS*.

Comparing the chromosomal region analyzed in our study to the canine genome sequence, the genes orthologous to HSA4 and BTA6 were discovered on canine chromosomes 3, 13, and 32 (CFA3,13, and 32, [38]). However, several breakpoints differing from other species were found. The interval ranging from near *NUP54 *to *CENPE/FLJ20032 *on HSA4 is completely located on CFA32 in an identical order. This interval also represents the longest syntenic gene interval transmitted without any breaks. An additional break was observed near *KIAA1276*, which was also detected on chicken GGA4. The segment involving genes from *KIAA1276 *to *LBD2 *is located on CFA3 without any break between *LAP3 *and *QDPR. *This is analogous to the mouse and rat chromosomes MMU5 and RNO14, but in contrast to BTA6. In addition, the interval comprising *MLR1 *to *UGDH *was inversely mapped on CFA3. The fragment from *KIAA1102 *to *IL8*, which is attached to *UGDH *on HSA4, appeared on CFA13.

From Zoo-FISH experiments, genetic, and RH mapping studies, it is known that the porcine chromosome syntenic to HSA4 and BTA6 is chromosome 8 (SSC8, e.g., [39,40]), whereas in horse the corresponding chromosomal region was identified on chromosomes 2 and 3 (ECA2 and ECA3, [41]). Due to the lower degree of details in gene-based mapping information from the currently available porcine and equine maps, accurate subchromosomal breakpoints and boundaries could not be retrieved for the construction of corresponding comparative maps. The proportion of genes mapped on the orthologous chromosomes is relatively small and, consequently, not sufficient to pursue the positions of the genes that have been mapped on our RH map of BTA6. For pig, the information we retrieved from several RH and linkage maps indicates that most of the genes from HSA4 are conserved on SSC8. In the recently published BAC-based comparative human-porcine physical map [42], 6 conserved syntenic regions from HSA4 were placed on SSC8 indicating that there a significant higher number of rearrangements in gene order exists compared to the human- bovine comparative map.

The analysis of the homologous chromosomal regions between species based on available sequence data and annotation of genes revealed the occurrence of gene deserts defined as megabase-size genomic segments devoid of protein-coding genes. On HSA4, which is populated with gene deserts corresponding to approximately 35 % of the length of the whole chromosome [43], there exists one outstanding region spanning about 8.5 Mb on HSA4p15.1, only separated by *PCDH7*. The second gene desert interval is located near the pericentromeric segment on HSA4q12-q13, where *LPHN3 *is flanked on both sides by gaps of about 4 and 3 Mb, respectively, without annotated protein-coding genes. When compared to genome sequences from dog, mouse, rat and chicken, the overall architecture of the gene desert region around *PCDH7 *was found to be conserved in mammals and birds including the flanking genes [44].

The complete genome sequence assembly of the bovine syntenic region of BTA6 is not yet available. Therefore, our high-resolution gene-based RH map of the targeted BTA6 was covered with a high density of anonymous loci in these gene-poor regions. Anchoring of a substantial number of the sequences of these anonymous loci onto HSA4 (see Additional file 2) enabled us to infer that the overall architecture of these gene deserts seems to be maintained also on BTA6.

Taken together, most of the rearrangements detected between the human HSA4 region and the chromosomal region on bovine BTA6, chicken GGA4, and most likely porcine SSC8 are intrachromosomal. In contrast, the respective chromosomal region in rodent, canine, and equine genomes, is transferred onto two or three chromosomes. Thus, our observations have suggested a higher number of conserved segments between the bovine, human, chicken and pig genomes than between the bovine and the second species group. This implies that the organization of the human genome regarding chromosomal synteny is closer to that of the cattle, chicken and pig than that of the mouse, rat, dog, and horse.

## Conclusion

In conclusion, the high-resolution RH map for the defined region of BTA6 presented here integrates anonymous markers, ESTs, and genes from currently available bovine linkage and RH maps as well as a high number of comparative anchor loci derived from orthologous HSA4. Although a number of links to the currently existing genetic, cytogenetic, and RH maps were possible, a multitude of contigs and scaffolds of the available bovine genome sequence resources still have to be anchored and/or oriented on the chromosomes. In this context, our high-resolution gene-enriched RH map will enable a great improvement of genome sequence assembly of BTA6 by linking the physical mapping data with sequence information from the preliminary sequence contig assembly of this chromosome. With a resolution of 1 locus/300 kb, our RH map will provide a valuable platform to guide the comprehensive high-quality assembly of the genome sequence and gene annotation on BTA6.

Furthermore, mapping a large number of genes on BTA6 and cross-referencing them with map locations in corresponding syntenic multi-species chromosome segments; our RH map will refine synteny segments at a high-resolution level to decipher mammalian chromosome evolution.

Finally, the connections between available physical and linkage maps, including cross-species comparative maps and bovine genome sequence provide a resource for gene-based fine mapping of QTL by increasing efficiency of identifying genes and causal mutations affecting animal performance. Connecting animal phenotypes associated with the QTL anchored on genomic sequence level with putative underlying genes would accelerate the identification of sequence polymorphisms and gene variants and the development of SNP markers for validation of association substantially.

## Methods

### Selection of markers and genes specific to BTA6

In addition to the 46 anonymous markers included in our previous BTA6 RH map [9], 32 markers from the recently published bovine genetic map [25] and 1 sheep marker (LSCV43) were selected to fill in defined subchromosomal intervals poorly covered. Moreover, 31 novel anonymous targeted sequence tagged sites (STS: *FBNS1-FBNS11, FBNS13-FBNS17, FBNS19-FBNS25, FBNS27-FBNS34*) that have been isolated from a microdissection library specific for the targeted BTA6 region [35], 10 BAC end sequences (BES) and 2 microsatellite sequences derived from 9 BAC clones (see Additional file 3) were integrated (see Additional file 1, labeled in green). Chromosomal location of targeted chromosome region specific markers was confirmed by synteny mapping using the INRA cattle-hamster somatic cell hybrid panel [45]. Furthermore, a total of 115 genes and ESTs were mapped on our high-resolution RH map. Therefore, in addition to the 25 loci included in our initial RH map for BTA6 [9], 27 genes and ESTs that all have been previously assigned to BTA6 [6,21,29] were integrated. Moreover, to enable identification of bovine sequences homologous to genes from the HSA4 chromosomal region, BLASTN analysis against the bovine genome resources (AAFC0200000000) [28] has been performed with default parameters using 60 mRNA reference sequences of genes located in the targeted interval of HSA4p16.3-HSA4q26 [46]. In addition, 3 bovine chromosome-region specific ESTs (*FBNE1-FBNE3*) known to be located on BTA6 from our unpublished results were included. Whereas *FBNE1 *displayed sequence similarity to human *HTN1*, with *FBNE2 *and *FBNE3 *no homologous human genes and ESTs were identified. The 63 novel genes and ESTs mapped on BTA6 without previous mapping information from published bovine physical maps are marked in red in Additional file 1. Homologous bovine EST sequences have been aligned to the corresponding human mRNA and genome sequences in order to identify the region of sequence homology, which had to be considered for primer design.

*De novo *primer pairs were designed using the OLIGO 5.0 program package (National Biosciences, Plymouth, MN, USA). Primers were preferably designed from ESTs revealing similarity to 3' untranslated regions of genes. If bovine ESTs showed similarity to the coding regions of the corresponding human genes, primers were designed within exons. Primers were tested for successful PCR amplification on bovine genomic DNA. In general, successful amplification was defined as a single amplicon of the predicted size with bovine DNA as visualized on agarose gel electrophoresis, but absence of the amplification product with genomic hamster DNA as a control. To verify that primers amplified the targeted gene, bovine PCR products were sequenced using PCR primers. Detailed information on primer sequences and PCR conditions is summarized in Additional file 1.

### Radiation hybrid screening

RH typing has been performed on the 12,000 rad whole genome cattle-hamster radiation hybrid (12 k WG-RH) panel [7]. Standard PCR was implemented as touch-down procedure as described previously [9]. PCR reactions were carried out in duplicate in a final volume of 10 μl including 50 ng hybrid cell line DNA, 4 pmol primers, 200 μM dNTPs, 0.25 units Taq DNA Polymerase (Promega, Madison, WI, USA) in 10 mM Tris-HCl pH 9.0, 50 mM KCl, 0.1% Triton X-100, and 1.5 mM MgCl_2_. Generally, PCR products were size-separated on agarose gels stained with ethidium bromide. PCR fragments of loci faintly amplified were analyzed on polyacrylamide gels visualized by silver staining [35]. To better discriminate between bovine and hamster amplicons, SSCP analysis was applied for two loci (*PDCH7, TXK*). For this purpose, PCR products were separated by running on 0.5× MDE gels (Biozym Scientific GmbH, Hess. Oldendorf, Germany) at 18°C in 0.6× TBE, pH 8.0, including 5 % glycerol, and visualized by silver staining.

Scoring of results was performed twice in two independent tests. In case of conflicting results, loci were retyped and reanalyzed. Any remaining discrepancies were scored as ambiguous.

### Radiation hybrid map construction

The RH map construction based on the analysis of the co-retention pattern of the loci included using the RHMAP program package version 3.0 [47]. The program RH2PT provided data description, e.g., retention rates and assembly of linkage groups based on twopoint analyses of loci. Multipoint programs RHMINBRK (calculation of map order with minimal number of obligatory chromosomal breaks) and RHMAXLIK (calculation of map order with maximized likelihood given the retention pattern) were applied for map construction under the equal retention model. Characteristics of the maps (locus order, number of obligate breaks) generated by the two multipoint programs were compared.

Starting point for map construction was the largest linkage group at twopoint lodscore (LOD) 16 comprising 196 loci with single retention patterns. Taking loci from this linkage group, an initial map was calculated applying a threshold of multipoint LOD 3. Further loci joining the LOD 16 linkage group at LOD 12, 10, and 8 were added sequentially, again applying a threshold of multipoint LOD 3. The resulting framework map was used as a starting point to add all remaining loci not entering the framework map to generate a comprehensive map displaying the highest likelihood of locus order. To assess the quality of the determined locus order the most likely order of loci within all LOD 16 (20 groups) and LOD 12 linkage groups (8 groups) was determined and compared to the final order of the comprehensive linkage group for LOD 8. Additionally, analogous to the "ripple" procedure to the RHMAPPER program [48], the most likely order for a sliding frame of eight loci was calculated along the chromosome.

At twopoint LOD 8, two groups of loci (*MAPK10*-*BMC4203*, *FGFBP1*-*LRPAP1*) did not enter the major linkage group of the 196 loci, but established two separate linkage groups at twopoint LOD 8 or 6, respectively. A loci order at multipoint LOD 3 and the most likely order of loci were determined within each of these two groups. Additionally, loci of these linkage groups were added to the final order of the major LOD 8 linkage group. Genes included in these two groups revealed a high twopoint LOD to two different anonymous markers with highly confirmed position on published BTA6 maps (*BMC4203 *and *BM2320*, respectively). The derived chromosomal order of these groups was in agreement with this mapping information and with the localization of ESTs homologous to *ACOX3 *and *LRPAP1 *on the SunbRH panel [6]. Thus, the information on chromosomal order was used to include the two groups for the final calculation of the physical map distances of our RH map.

### BAC screening, sequencing and localization

PCR-based screening of DNA superpools was performed on the BBI_B750 bovine BAC library [49] available at the Resource Center, Berlin [50] using sequences from 7 anonymous markers (*FBN13, FBN14, FBN20, FBNS10, BM143, BM3026, and OARJMP36) *and 1 gene-derived sequence (*PPARGC1A*). Specificity of the identified BAC clones was verified by direct sequencing with primers used for BAC library screening. The chromosomal location of the BACs was determined by Fluorescence *in situ *hybridization [51]. Identified BAC clones (see Additional file 3) were end-sequenced with T7 and SP6 primers.

### Sequence similarity search on the bovine and human genome sequences

Sequences from loci applied for RH mapping on BTA6 but not included in the current sequence assembly map of BTA6 at NCBI (build 2.1, [27]) were used for sequence similarity screening in the currently available 6× coverage draft sequence of the bovine genome [28]. The similarity search was performed in the database [genome (reference only)] using the Basic Local Alignment Search Tool (MegaBLAST) with default parameters.

Sequences from bovine anonymous markers (microsatellite markers, targeted newly developed anonymous markers, and BES) mapped on the BTA6 RH map were applied to similarity search against human genomic sequences [32] using the Basic Local Alignment Search Tool (BLASTN) to place them *in silico *on syntenic chromosomal regions of the human genome. For this sequence similarity screening we selected the NCBI database Homo sapiens [ORGN] [32] and used default parameters. Only for the search with short microsatellite sequences, the expect value was set to 100. The information about comparative placement of the bovine anonymous sequences on identified human BAC contigs was retrieved from mapviewer NCBI (build 35.1 version) of the human genome assembly [46].

If the sequences of the anonymous bovine loci did not match a homologous sequence on the human genome by direct similarity search, the bovine whole-genome sequences (WGS) containing the targeted anonymous sequences were used as intermediary reference sequences. For this purpose, the anonymous sequences, which did not retrieve human homologous sequences by direct similarity search on the human genome sequence, were blasted first against the bovine genomic sequence database [WGS contigs] with default parameters [28]. In the second step, the bovine WGS identified in the NCBI sequence archive were blasted against the human genomic sequence as described above for direct bovine-human similarity search.

Generally, each match found in the sequence databases was manually curated. Matches were excluded when revealing less than 95 % identity for within species blast searches and less than 80 % identity across species. Alignments with less than 30 consecutive nucleotides and matches containing annotations indicating the presence of repetitive elements not detected by the Repeatmasker filter were eliminated.

### Comparative map construction

To construct the multi-species comparative maps, gene positions mapped on the BTA6 12 k WG-RH map were aligned with corresponding genome map positions on orthologous chromosomal regions of human, mouse, rat, chicken, and dog. Gene mapping information was retrieved from the species genome sequence maps available at the NCBI mapviewer database [33]: human chromosome 4 (HSA4, NCBI build 35.1), mouse chromosomes 5, 6, and 3 (MMU5, 6, and 3; NCBI build 34.1), rat chromosomes 14, 2, and 4 (RNO14, 2, and 4; NCBI build 3.1), dog chromosomes 3, 13, and 32 (CFA3,13 and 32, NCBI build1.1), and chicken chromosome 4 (GGA4, NCBI build 1.1).

## Authors' contributions

RW developed RH markers, performed RH mapping, BAC library screening and BLAST analyses on genome sequence databases, and constructed the comparative maps. CK performed the statistical analysis of the RH data, calculated and constructed the BTA6 RH map. TG performed FISH mapping of BAC clones. PL provided the somatic cell hybrid panel and did chromosomal assignments of newly developed markers. JEW developed and provided the 12 k WG-RH panel. RW and CK wrote the manuscript draft. All authors read and approved the final manuscript.

## Supplementary Material

Additional File 2 Excel spreadsheet containing mapping information and homology of sequences mapped on the 12 k WG-RH map of BTA6. *Entitled*: **Mapping information and homology of sequences mapped on the 12 k WG-RH map of BTA6**. The order of loci is given according to the 12 k WG-RH map presented in this study. (1) Loci in cells highlighted light green represent framework markers in the 12 k WG-RH map. (2) Accession numbers in bold blue: Homology of BAC contigs from human chromosome 4 (HSA4) was identified by blasting the bovine anonymous sequences directly against the human genome sequence. Accession numbers in underlined blue: Homology on HSA4 was obtained using identified bovine whole-genome sequences (WGS) sequences containing the sequences of targeted anonymous loci as intermediary sequence for blasting against the human genome sequence. Empty cells: no significant homology was found. (3) Loci with identical retention pattern and position on the 12 k WG-RH map of BTA6 are indicated in green. (4) Identified bovine WGS containing the sequences of targeted anonymous loci, which did not find homologous human sequences by direct sequence similarity search. WGS were used as intermediary sequence for blasting against the human genome sequence. (5) Sequence contigs given in black were retrieved from the available sequence map of BTA6 at NCBI (build 2.1, [27]). Sequence contigs marked in bold red represent new assignments of loci to BTA6 specific sequence contigs. Sequence contigs marked in ordinary red letters are assigned on the currently available bovine STS map. Sequence contigs given in blue indicate new loci assignments to unplaced bovine contigs. Cells highlighted in yellow indicate sequence contigs that have been assigned to other chromosomes than BTA6. Empty cells: no significant homology was found. Click here for file

Additional File 1 Excel spreadsheet containing loci mapped on the 12 k WG-RH map of BTA6, primer sequences and PCR conditions. *Entitled*: **Loci mapped on BTA6, primer sequences and PCR conditions**. Loci given in red represent novel genes and ESTs mapped on BTA6 without previous mapping information on bovine physical maps. Loci given in green represent newly developed targeted anonymous markers (STS) and BAC end sequences (BES). Loci indicated in black were previously mapped on BTA6 [9,21,24,25,52-58]. Accession numbers (AccNo) indicate sequences used for primer design. Black AccNo: bovine sequence, Blue AccNo: human sequence. Size: amplicon length in bp, TA: annealing temperature in °C. Click here for file

Additional File 3 Excel spreadsheet containing BTA6-specific BAC clones and BAC end sequences. *Entitled*: ***BTA6-specific BAC clones and BAC end sequences.*** Click here for file
